# Supplementary material for: Survival Benefits of Chemotherapy for Patients with Advanced Pancreatic Cancer in A Clinical Real-World Cohort
Source: Cancers (Basel). 2019 Sep 7;11(9):1326. doi: 10.3390/cancers11091326 (PMC6769947; doi:10.3390/cancers11091326)
Supplement: Supplementary file 1 [file cancers-11-01326-s001.zip › Figure S1.pdf]

Figure S1

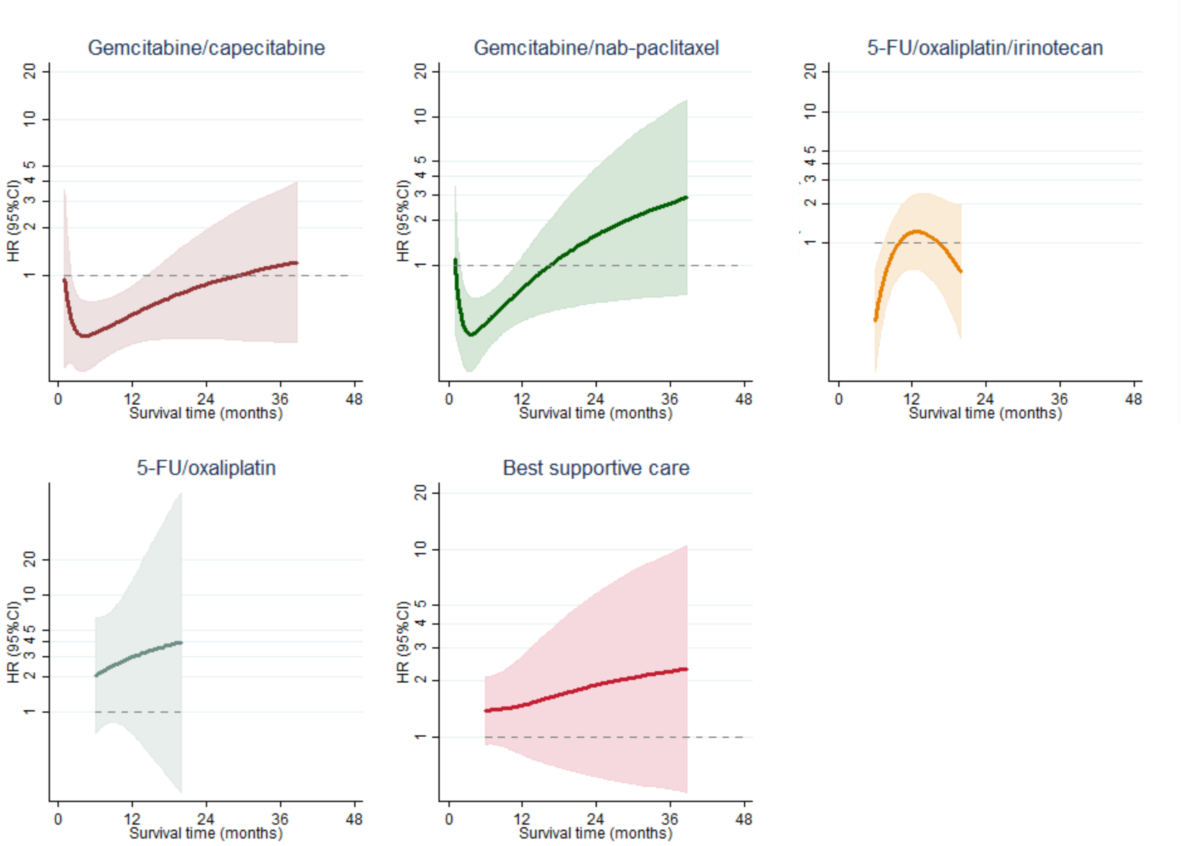

Figure S1 – Flexible parametric survival model of the HR for death according to first-line chemotherapy compared to monotherapy with gemcitabine among pancreatic cancer patients without previous surgery. The models were adjusted for age, sex, BMI, alcohol consumption, smoking status, diabetes, tumor stage, tumor grade, ECOG score, and CA19-9 level.
