# Supplementary material for: Survival Benefits of Chemotherapy for Patients with Advanced Pancreatic Cancer in A Clinical Real-World Cohort
Source: Cancers (Basel). 2019 Sep 7;11(9):1326. doi: 10.3390/cancers11091326 (PMC6769947; doi:10.3390/cancers11091326)
Supplement: Supplementary file 1 [file cancers-11-01326-s001.zip › Table S1.pdf]

Table S1

Table S1: Baseline characteristics of patients according to second-line therapy									
**Characteristic**	**Gemcitabine (N = 27)**	**Gemcitabine/ nab-paclitaxel (N = 23)**	**5-FU/oxaliplatin (N = 53)**	**5-FU/ irinotecan (N = 19)**	**5-FU; capecitabine (N = 13)**	**Other (N = 13)**	**Best supportive care (N = 147)**	**All patients (N = 295)**	**P-value**
First-line regimen – no. (%)									
Gemcitabine	–	2 (8.7)	15 (28.3)	1 (5.3)	7 (53.8)	4 (30.8)	96 (65.3)	125 (42.4)	.000
Gemcitabine/capecitabine	10 (37)	3 (13)	20 (37.7)	1 (5.3)	2 (15.4)	4 (30.8)	14 (9.5)	54 (18.3)	
Gemcitabine/nab-paclitaxel	7 (25.9)	–	15 (28.3)	12 (63.2)	2 (15.4)	1 (7.7)	15 (10.2)	52 (17.6)	
5-FU/oxaliplatin/irinotecan	6 (22.2)	15 (65.2)	2 (3.8)	1 (5.3)	–	2 (15.4)	3 (2)	29 (9.8)	
5-FU/oxaliplatin	4 (14.8)	2 (8.7)	–	4 21.1)	1 (7.7)	–	16 (10.9)	27 (9.2)	
Other	–	1 (4.3)	1 (1.9)	–	1 (7.7)	2 (15.4)	3 (2)	8 (2.7)	
Sex – no. (%)									
Female	14 (51.9)	6 (26.1)	20 (37.7)	9 (47.4)	9 (69.2)	6 (46.2)	67 (45.6)	131 (44.4)	.231
Male	13 (48.1)	17 (73.9)	33 (62.3)	10 (52.6)	4 (30.8)	7 (53.8)	80 (54.4)	164 (55.6)	
Age at diagnosis – years									
Mean (range)	64.3 (51.3–77)	62 (39.7–76.1)	64.9 (38–81.6)	66.2 (55.5–80.5)	70.3 (57.3–82.8)	66.6 (48.9–76.4)	69.7 (46.6–83.8)	68.3 (38–83.8)	.000
Body mass index – no. (%)*									
≤ 18.4	2 (7.4)	–	3 (5.7)	–	1 (7.7)	–	11 (7.5)	17 (5.8)	.694
18.5–29.9	23 (85.2)	22 (95.7)	49 (92.5)	19 (100)	11 (84.6)	13 (100)	125 (85)	262 (88.8)	
≥ 30.0	2 (7.4)	1 (4.3)	1 (1.9)	–	1 (7.7)	–	10 (6.8)	15 (5.1)	
ECOG performance status – no.(%)*									
0	9 (33.3)	13 (56.5)	25 (47.2)	7 (36.8)	7 (53.8)	3 (23.1)	37 (25.2)	101 (34.2)	.000
1	11 (40.7)	9 (39.1)	19 (35.8)	9 (47.4)	3 (23.1)	7 (53.8)	79 (53.7)	137 (46.4)	
2	1 (3.7)	1 (4.3)	6 (11.3)	1 (5.3)	2 (15.4)	1 (7.7)	29 (19.7)	41 (13.9)	
≥3	4 (14.8)	–	1 (1.9)	–	1 (7.7)	2 (15.4)	2 (1.4)	10 (3.4)	
Diabetes – no. (%)*†									
yes	5 (18.5)	5 (21.7)	8 (15.1)	3 (15.8)	5 (38.5)	4 (30.8)	47 (32)	77 (26.1)	.167
no	22 (81.5)	18 (78.3)	44 (83)	16 (84.2)	8 (61.5)	8 (61.5)	100 (68)	216 (73.2)	
Alcohol abuse – no. (%)*†									
yes	–	1 (4.3)	1 (1.9)	1 (5.3)	2 (15.4)	–	8 (5.4)	13 (4.4)	.495
no	23 (85.2)	22 (95.7)	45 (84.9)	18 (94.7)	11 (84.6)	10 (76.9)	127 (86.4)	256 (86.8)	
former	2 (7.4)	–	1 (1.9)	–	–	1 (7.7)	4 (2.7)	8 (2.7)	
Smoking – no. (%)*†									
yes	6 (22.2)	2 (8.7)	6 (11.3)	–	2 (15.4)	1 (7.7)	33 (22.4)	50 (16.9)	.403
no	9 (33.3)	12 (52.2)	21 (39.6)	8 (42.1)	6 (46.2)	6 (46.2)	60 (40.8)	122 (41.4)	
former	10 (37)	9 (39.1)	19 (35.8)	11 (57.9)	5 (38.5)	4 (30.8)	47 (32)	105 (35.6)	
Primary tumor location – no. (%)*									
Head	15 (55.6)	14 (60.9)	27 (50.9)	10 (52.6)	7 (53.8)	5 (38.5)	91 (61.9)	169 (57.3)	.382
Body	4 (14.8)	7 (30.4)	10 (18.9)	2 (10.5)	1 (7.7)	1 (7.7)	24 (16.3)	49 (16.6)	
Tail	2 (7.4)	2 (8.7)	3 (5.7)	3 (15.8)	2 (15.4)	2 (15.4)	14 (9.5)	28 (9.5)	
Overlapping	5 (18.5)	–	10 (18.9)	2 (10.5)	1 (7.7)	3 (23.1)	14 (9.5)	35 (11.9)	
Ampulla of Vater	–	–	3 (5.7)	1 (5.3)	1 (7.7)	2 (15.4)	4 (2.7)	11 (3.7)	
Metastasization – no. (%)‡									
Non-local lymph nodes	1 (3.7)	1 (4.3)	4 (7.5)	2 (10.5)	1 (7.7)	2 (15.4)	7 (4.8)	18 (6.1)	.716
Liver	9 (33.3)	10 (43.5)	27 (50.9)	12 (63.2)	8 (61.5)	5 (38.5)	69 (46.9)	140 (47.5)	.433
Lung	6 (22.2)	3 (13)	6 (11.3)	1 (5.3)	3 (23.1)	1 (7.7)	26 (17.7)	46 (15.6)	.550
Peritoneum	4 (14.8)	5 (21.7)	7 (13.2)	4 (21.1)	2 (15.4)	4 (30.8)	14 (9.5)	40 (13.6)	.267
Other	–	–	2 (3.8)	–	1 (7.7)	–	15 (10.2)	18 (6.1)	.156

cont.	*Gemcitabine*	*Gemcitabine/ nab-paclitaxel*	*5-FU/oxaliplatin*	*5-FU/ irinotecan*	*5-FU; capecitabine*	*Other*	*Best supportive care*	*All patients*	*P-value*
No. of tumor sites – no. (%)‡									
1	13 (48.1)	8 (34.8)	16 (30.2)	5 (26.3)	5 (38.5)	4 (30.8)	64 (43.5)	227 (38.2)	.514
2	10 (37)	13 (56.5)	32 (60.4)	12 (63.2)	5 (38.5)	7 (53.8)	61 (41.5)	266 (44.7)	
≥3	4 (14.8)	2 (8.7)	5 (9.4)	2 (10.5)	3 (23.1)	2 (15.4)	22 (15)	100 (16.8)	
Morphology – no. (%)*									
Adenocarcinoma	21 (77.8)	19 (82.6)	48 (90.6)	17 (89.5)	12 (92.3)	12 (92.3)	124 (84.4)	253 (85.8)	.404
Other	–	1 (4.3)	–	1 (5.3)	–	1 (7.7)	2 (1.4)	5 (1.7)	
Carbohydrate antigen 19–9 – kE/l*§									
Median (IQR)	1390 (95–7609)	306 (120–2932.5)	965 (191–4719.5)	849.5 (68.8–4900)	593 (78.5–2439.3)	80 (16–2949)	574 (131–3751.8)	708 (101.8–3855.3)	.357
Surgery									
Tumor resection – no. (%)	2 (7.4)	3 (13)	7 (13.2)	5 (26.3)	4 (30.8)	1 (7.7)	39 (26.5)	61 (20.7)	.085
Median time to relapse – months (IQR)	15.4 (14.2–16.5)	13.1 (9.6–21.5)	3.8 (0.9–7.4)	7.7 (5–10.3)	10.5 (8.2–12.5)	5.3 (5.3–5.3)	11.8 (8.4–15.4)	9.4 (5.8–14.2)	.067
Adjuvant chemotherapy – no. (%)¶									
Total	2 (7.4)	3 (13)	3 (5.7)	5 (26.3)	4 (30.8)	1 (7.7)	28 (19)	46 (15.6)	.089
Completed	2 (7.4)	2 (8.7)	2 (3.8)	3 (15.8)	2 (15.4)	–	22 (15)	33 (11.2)	
Interrupted	–	1 (4.3)	1 (1.9)	2 (10.5)	2 (15.4)	1 (7.7)	6 (4.1)	13 (4.4)	
Interventions at diagnosis – no.(%)									
ERCP/PTC	14 (51.9)	12 (52.2)	23 (43.4)	8 (42.1)	7 (53.8)	5 (38.5)	71 (48.3)	140 (47.5)	.919
Exploration	2 (7.4)	4 (17.4)	6 (11.3)	2 (10.5)	1 (7.7)	3 (23.1)	12 (8.2)	30 (10.2)	.248

*Percent missing up to 100 = no information available. †According to physician's note. ‡Percent missing up to 100 = no distant metastases. § Reference interval <34 kE/l. Abbreviations: ECOG = Eastern Cooperative Oncology Group; ERCP = Endoscopic retrograde cholangiopancreatography; IQR = Interquartile range; n/a = not applicable; PTC = Percutaneous transhepatic cholangiography.
