# Supplementary material for: Survival Benefits of Chemotherapy for Patients with Advanced Pancreatic Cancer in A Clinical Real-World Cohort
Source: Cancers (Basel). 2019 Sep 7;11(9):1326. doi: 10.3390/cancers11091326 (PMC6769947; doi:10.3390/cancers11091326)
Supplement: Supplementary file 1 [file cancers-11-01326-s001.zip › Table S2.pdf]

Table S2

Table S2: Stratification of overall survival (OS) by metastatic stage

Treatment		Total	Non-metastatic disease	Metastatic disease
Gemcitabine	Patients (N)	185*	65	119
	Median OS – months (95 % CI)	6.6 (5.5–7.7)	8.2 (6.6–9.7)	5.7 (4.8–6.6)
Gemcitabine/ capecitabine	Patients (N)	60	22	38
	Median OS – months (95 % CI)	10.6 (7.8–13.3)	12.1 (8.8–15.4)	8.7 (4.6–12.9)
Gemcitabine/ nab-paclitaxel	Patients (N)	66	6	57
	Median OS – months (95 % CI)	9.8 (7.9–11.8)	9.2 (3.4–15)	9.8 (7.7–11.9)
5-FU/oxaliplatin/ irinotecan	Patients (N)	31	15	16
	Median OS – months (95 % CI)	9.9 (8.1–11.7)	9.9 (7–12.8)	10.3 (8.6–12)
5-FU/oxaliplatin	Patients (N)	35	12	23
	Median OS – months (95 % CI)	5.8 (4.5–7)	8.4 (4–12.9)	5.2 (3.6–6.8)

*For four patients, no information on the M-stage was available
