# Supplementary material for: Survival Benefits of Chemotherapy for Patients with Advanced Pancreatic Cancer in A Clinical Real-World Cohort
Source: Cancers (Basel). 2019 Sep 7;11(9):1326. doi: 10.3390/cancers11091326 (PMC6769947; doi:10.3390/cancers11091326)
Supplement: Supplementary file 1 [file cancers-11-01326-s001.zip › Table S3.pdf]

Table S3

Table S3: Hazard ratio for death according to treatment and stratified by previous pancreatic surgery

Treatment	With surgery			Without surgery§	
	Number of patients	HR (95% CI)#		Number of patients	HR (95% CI)#
Gemcitabine	31	1 (ref)		154	1 (ref)
Gemcitabine/capecitabine	5	0.48 (0.15–1.60)		55	0.54 (0.37–0.78)**
Gemcitabine/nab-paclitaxel	8	0.55 (0.18–1.70)		58	0.54 (0.38–0.79)**
5-FU/oxaliplatin/irinotecan	2	0.53 (0.06–4.35)		29	0.48 (0.29–0.81)**
5-FU/oxaliplatin	30	1.15 (0.59–2.27)		5	1.90 (0.74–4.85)
Best supportive care	62	2.33 (1.20–4.50)*		144	2.81 (2.06–3.82)***

HR, hazard ratio; CI, confidence interval.
*, p < 0.05; **, p < 0.01; ***, p < 0.001.
#, multivariate logistic regression; models were adjusted for age, sex, BMI, alcohol consumption, smoking status, diabetes, tumor stage, tumor grade, ECOG score, and CA19-9 level.
§, this model violated the proportional-hazard assumption so additional flexible parametric survival models were used.
